# Active Suspension Control Strategy of Multi-Axle Emergency Rescue Vehicle Based on Inertial Measurement Unit

**DOI:** 10.3390/s21206877

**Published:** 2021-10-16

**Authors:** Qinghe Guo, Dingxuan Zhao, Xiaolong Zhao, Zhenxing Li, Xiaobo Shi

**Affiliations:** 1 School of Mechanical Engineering, Yanshan University, Qinhuangdao 066004, China; qinghekwok@126.com (Q.G.); zxlysu@139.com (X.Z.); lizhenxing19961227@163.com (Z.L.); 2 Key Laboratory of Special Carrier Equipment of Hebei Province, Yanshan University, Qinhuangdao 066004, China; shizipi_009@stumail.ysu.edu.cn; 3 School of Electrical Engineering, Yanshan University, Qinhuangdao 066004, China

**Keywords:** multi-axle vehicle, electro-hydraulic servo, active suspension, control strategy

## Abstract

Active suspension control strategies are a top priority in active suspension system. The current research on active suspension control strategies is mostly focused on two-axle vehicles, and there is less research investigating multi-axle vehicles. Additionally, their effective implementation is dependent on accurate mathematical models, and most of them adopt force feedback control, which is vulnerable to external interference. To solve these problems, this paper proposes an active suspension control strategy based on Inertial Measurement Unit. The multi-axle emergency rescue vehicle is made to be equivalent to a 3-degrees-of-freedom parallel mechanism by using the method of grouping and interconnecting the suspension units of the whole vehicle. The attitude change of the vehicle body was transformed into the servo actuator’s displacement by solving the inverse solution of the parallel mechanism position and the action of the servo actuator was driven in reverse according to the displacement obtained. In this way, the vehicle body attitude can be compensated, and the ride comfort and the handling stability of the vehicle can be improved. To verify the effectiveness of the control strategy proposed, the three-axle six vehicle was taken as the research object, the position inverse solution of its equivalent 3-degrees-of-freedom parallel mechanism was deduced, and a high-pass filter was designed. The three-axle vehicle experiment platform integrating active suspension and hydro-pneumatic suspension was built, and the gravel road and slope road experiments were carried out and the results compared with those obtained with hydro-pneumatic suspension. The experiment results showed that, compared with hydro-pneumatic suspension, the active suspension control strategy based on Inertial Measurement Unit proposed in this paper can not only stabilize the body attitude, but also effectively suppress body vibration, improving the ride comfort and handling stability of the vehicle significantly.

## 1. Introduction

As the main force of post-disaster rescue, emergency rescue vehicles are indispensable rescue equipment. Because of their advantages in terms of large transportation volume, low transportation cost, strong bearing capacity and high road friendliness, multi-axle emergency rescue vehicles have become a key research object in the field of rescue vehicles. However, the current chassis of multi-axle emergency rescue vehicles are generally refitted from the chassis of passenger cars or trucks, and their suspensions is usually passive suspension. The structural parameters of passive suspension are fixed and cannot be adjusted in real time according to the driving road conditions. Generally, the terrain environment of the area in which the rescue is to be performed is more complex than a general road. When multi-axle emergency rescue vehicles equipped with passive suspension drive in such areas, this will inevitably lead to strong bumps, vibration and large attitude changes of the vehicle body. The strong bumps, vibration and large changes in attitude can not only cause secondary injury to the wounded, but also affect the normal usage of on-board instruments and equipment. Therefore, passive suspension makes it difficult to ensure the ride comfort and handling stability of multi-axle emergency rescue vehicles, seriously limiting the off-road driving speed and rescue efficiency. With the development of electronic information technology, the application of active suspension in vehicle field has become possible. Active suspension can obtain the body motion state using sensors installed on the body, and actuators output the corresponding force or displacement in real time according to control signals from the active suspension. In this way, it is possible to meet the requirements of different working conditions on the suspension system characteristic parameters. If the active suspension is applied to multi-axle emergency rescue vehicle, its driving performance can be effectively improved.

The core of active suspension technology lies in the actuator and the control strategy. Actuators output force according to the instructions of the control strategy, and different control strategies will produce different suspension characteristics. Therefore, the key of active suspension technology is to select a control strategy that is able to provide good performance for the vehicle. In recent years, many experts and scholars have conducted extensive research on active suspension control strategies (ASCSs), and put forward a variety of advanced ASCSs.

Optimal control is an earlier control strategy that has been applied to active suspension. Lan and Yu [1] designed an LQG controller vehicle active suspension based on the half vehicle model, built the system model in MATLAB/Simulink, and carried out a comparative simulation with passive suspension. The simulation results showed that the active suspension with LQG controller effectively improved the ride comfort compared to passive suspension. On the basis of two different control methods (traditional method (CM) and acceleration correlation method (ADM)), Kumar et al. [2] designed a passenger car active suspension controller by using linear quadratic optimal control theory. The results showed that, compared with the passive suspension system, both the active CM system and the active ADM system are able to reduce seat acceleration to varying degrees, improving the ride comfort and road holding. Aiming at the non-linear problem of active suspension system, Khan et al. [3] proposed an improved half-car model control method. The input/output feedback linearization method was used to transform the nonlinear half-car model system into an equivalent linear system, and then an LQR controller was designed. The simulation results proved that the improved half-car model control method significantly improved the ride comfort of the vehicle. Uncertain parameters and external interference in the active suspension control system affect system performance. For this problem, Pang of Xi’an University of Technology, and Yan and Pan of Harbin Institute of Technology applied the adaptive control strategy to active suspension systems in [4,5,6], respectively, and achieved good control results. In addition to the uncertain parameters, there are also nonlinear problems in active suspension systems. Sliding mode control is a control strategy with strong ability to deal with nonlinear problems. Guan et al. [7] proposed a sliding mode control strategy that had two closed loops, where the outer loop considered the spring velocity of a skyhook reference model output as the tracking target and the inner loop regarded the desired force of the sliding mode solver as the tracking target. The simulation results showed that the proposed sliding mode control law was able to achieve accurate tracking of the desired force and improve the ride comfort. Instead of dividing the system into an actuator subsystem and a suspension subsystem, Xiao et al. [8] divided the active suspension system into a linear subsystem and a nonlinear subsystem, which greatly facilitated controller design. The sliding mode controller was created by specifying suitable sliding functions for the two subsystems respectively, and forcing the output of the nonlinear subsystem to track the desired fictitious input of the linear subsystem. The simulation results showed that the proposed control strategy was effective. Precup et al. [9] designed a method that focused on two-degrees-of-freedom fuzzy control system structures, and the suggested design method was validated by real-time experimental results using fuzzy controlled nonlinear DC drive-type laboratory equipment. Turnip et al. [10] developed a combination of skyhook and ground hook control-based magneto rheological lookup table technique which was used on the hybrid control for a quarter car, and the simulation indicated that the proposed hybrid control lookup table provided better vibration isolation capability than other methods. Precup et al. [11] developed the hybrid data-driven fuzzy ADRC algorithms and carried out comparative experiments to verify the effectiveness of the proposed control method.

Each control strategy has its own advantages in solving specific kinds of problems, but active suspension systems are highly complex. Relying on only one strategy will often take care of one thing while losing another. Therefore, the combination of two or more control strategies is often applied for the control of active suspension systems, for example, optimal sliding mode control [12], adaptive sliding mode control [13,14], fuzzy sliding mode control [15,16,17], adaptive neural network sliding mode control [18], preview fuzzy control [19], etc.

Although the advanced control strategies mentioned above have improved the performance of active suspension systems to varying degrees, there are still some problems regarding application. Firstly, the effective implementation of most control strategies is based on accurate suspension system models. In practice, the uncertainty of the parameters, the interference of the external environment and the nonlinearity of the system make it very difficult to obtain an accurate mathematical model, especially for multi-axle vehicles. As a result, most of them have remained in the theoretical simulation stage, with few of them having been put into practical engineering application. Secondly, most ASCSs are based on force feedback control, which requires the actuator to have better force tracking performance. However, for active suspension systems with electro-hydraulic servo actuators, pressure impact, pressure loss along the pipeline, and friction between piston and hydraulic cylinder wall cause great difficulties for force tracking, which is not conducive to the performance of active suspension systems. Compared to the force tracking control, displacement tracking control is less affected by external factors and is less difficult to realize. Therefore, the development of an ASCS based on displacement feedback could be considered. In addition, most ASCSs were developed for two axles, and the derivation process is relatively complicated, making them unsuitable for multi-axle vehicles. Therefore, for the control of the active suspension system of the multi-axle emergency rescue vehicles, it is necessary to further explore an ASCS that is economical and applicable.

When the multi-axle emergency rescue vehicle is driving on an unstructured road, the vehicle body shows vertical, roll and pitch motions under the excitation of the uneven road. The three motions are coupled and affect each other, resulting in changes in the position and attitude of the vehicle body [20]. The large change in body attitude is not only extremely unfavorable for the treatment of the wounded and the normal use of on-board instruments, but also results in poor ride comfort and handling stability of the vehicle. The control goal of the active suspension is to improve the ride comfort and handling stability of the vehicle, and the body attitude just can reflect the performance of these two aspects [21,22,23].

Aiming at the problems of the current ASCSs and considering the significance of body attitude control, an ASCS based on Inertial Measurement Unit (IMU) suitable for multi-axle vehicles is proposed in this paper. In this control strategy, firstly, the multi-axle emergency rescue vehicle was made equivalent to a 3-degrees-of-freedom (3-DOF) parallel mechanism by grouping and interconnecting the suspension system units of the whole vehicle; then, the change of the vehicle body attitude was transformed into the average displacement of each group of actuators by solving the inverse position solution of the parallel mechanism; and finally, according to the obtained average displacement, each group of actuators was reversed in order to realize the effective correction and compensation of the vehicle body attitude. In this way, the ride comfort and the handling stability of the multi-axle emergency rescue vehicle can be improved effectively.

The application of the ASCS proposed in this paper was not dependent on the vehicle dynamics model, thus effectively avoiding the adverse effects caused by the uncertainty of the system parameters. Moreover, in the ASCS proposed in this paper, the actuator is controlled by displacement feedback rather than force feedback. Compared with the force feedback control scheme used in other control strategies, the displacement feedback control scheme can reduce the influence of the external interference on the control accuracy of the system. Last but not least, the displacement control of the actuator was not the independent control of each actuator, but the integrated control with the average displacement of all actuators in the group as the control object. This displacement control method can reduce the control dimension and the control difficulty of the system. The ASCS proposed in this paper could make the vehicle body move along a straight line or arc, while the body attitude remains basically unchanged.

The remaining content of this paper is organized as follows. In Section 2, the control principle of ASCS based on IMU and the construction method of the equivalent 3-DOF parallel mechanism of the multi-axle vehicle are described. In Section 3, the equivalent 3-DOF parallel mechanism of the three-axis vehicle is constructed, the position inverse solution is deduced, and the high-pass filter is designed. In Section 4, the three-axis vehicle experiment platform integrating active suspension and hydro-pneumatic suspension is built, and the gravel road and slope road experiment are carried out. In Section 5, we summarize the paper and give the conclusions and the discussion.

## 2. Control Principle of the ASCS Based on IMU

**Figure 1:**
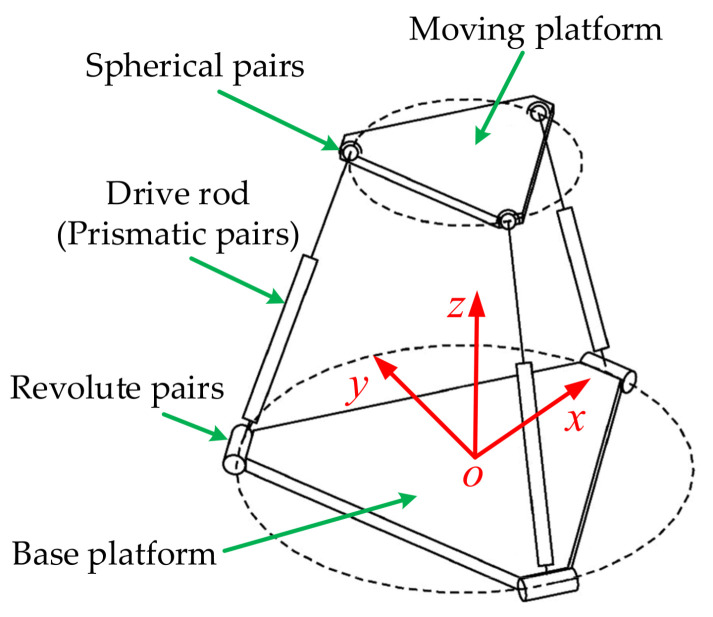
3-RPS parallel mechanism.

The 3-RPS parallel mechanism [24,25] is a typical representative of the spatial 3-DOF parallel mechanism. It is mainly composed of a moving platform, a base platform and three retractable drive rods (prismatic pairs) connecting the two platforms, as shown in Figure 1. The moving platform is connected with the driving rod through spherical pairs. The base platform is connected with the driving rod through revolute pairs. The 3-RPS parallel mechanism has two rotational degrees of freedom around the *x*-axis and around the *y*-axis, and one translational degree of freedom along the *z*-axis. When the three drive rods elongate or shorten according to the displacement instructions, the attitude of the moving platform changes in the space. When the attitude of the moving platform needs to be adjusted, the attitude of the moving platform is usually set first, and the displacements of the three driving rods are then obtained by solving the inverse solution [26]. The reason for this is that the inverse kinematics analysis of the parallel mechanism is simpler and more direct than the forward kinematics analysis.

When the multi-axis emergency rescue vehicle is driving on an uneven road, the vehicle body exhibits roll rotation, pitch rotation and vertical movement relative to the horizontal plane under the excitation of the road surface, which is the same as the movement of the 3-RPS parallel mechanism moving platform in space. Therefore, the multi-axle emergency vehicle can be considered to be equivalent to a 3-DOF parallel mechanism that is similar to the 3-RPS configuration, and we can use the attitude control method of the 3-RPS parallel mechanism as a reference to realize the attitude control of vehicle body.

**Figure 2:**
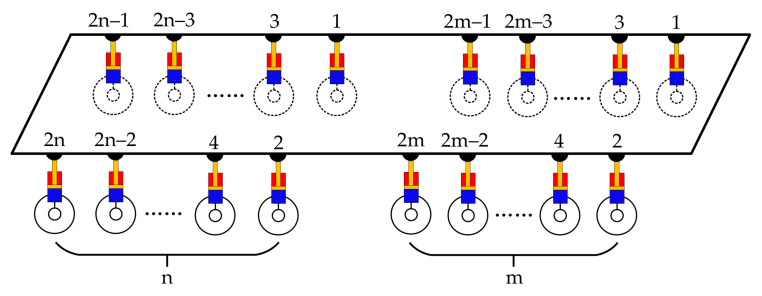
Multi-axle vehicle.

As can be seen from the 3-RPS parallel mechanism, the number of connection points between the driving rod and the base platform is 3, and the fulcrum number of the driving rod to the moving platform is also 3. However, for the multi-axle vehicle shown in Figure 2, the numbers of tire grounding points and support points of the suspension servo actuator cylinder on the vehicle body are both 2(m + n) (m and n are the number of the vehicle axles, and m ≥ 1, n ≥ 1), which is greater than 3. If the multi-axle vehicle is to be equivalent to a 3-DOF parallel mechanism, the multiple support points and the multiple tire grounding points of the vehicle need to be equivalently transformed to 3.

**Figure 3:**
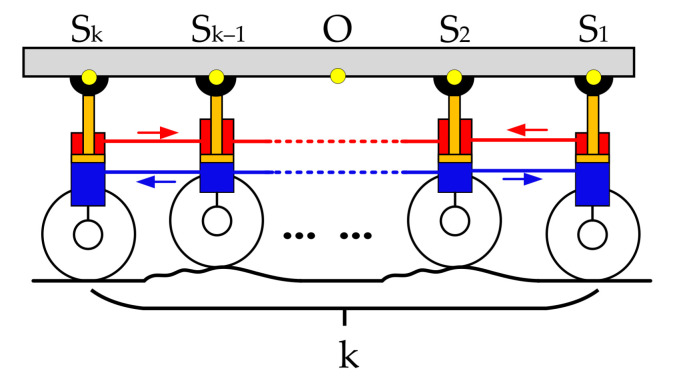
Hydro-pneumatic balance suspension of multi-axle vehicle.

During the driving process of multi-axle vehicles, uneven road surfaces lead to uneven axle load and even single axle overload, which seriously affects driving safety. To solve this problem, scholars from various countries have developed a variety of structural forms of balanced suspension. Among these, hydro-pneumatic balance suspension is widely favored in industry because it can ensure the uniform bearing capacity of each fulcrum without a precise balance bar system [27,28]. According to the structure and characteristics of hydro-pneumatic balanced suspension, if all of the rodless cavities and the rod cavities of a group of suspension cylinders are connected in turn, multiple support points (S_1_, S_2_…, S_k_) of the suspension cylinder on the vehicle body in the group can be equivalent to one support point (O), as shown in Figure 3. When the structural parameters of all the suspension cylinders in the group are the same, point O is located at the geometric center of all support points of the suspension cylinder on the vehicle body.

**Figure 4:**
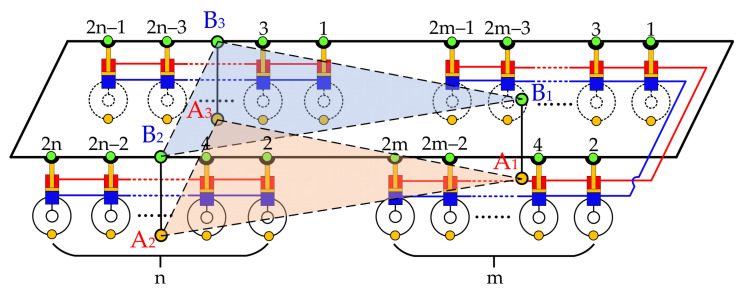
Schematic diagram of equivalent fulcrum of multi-axle vehicle.

**Figure 5:**
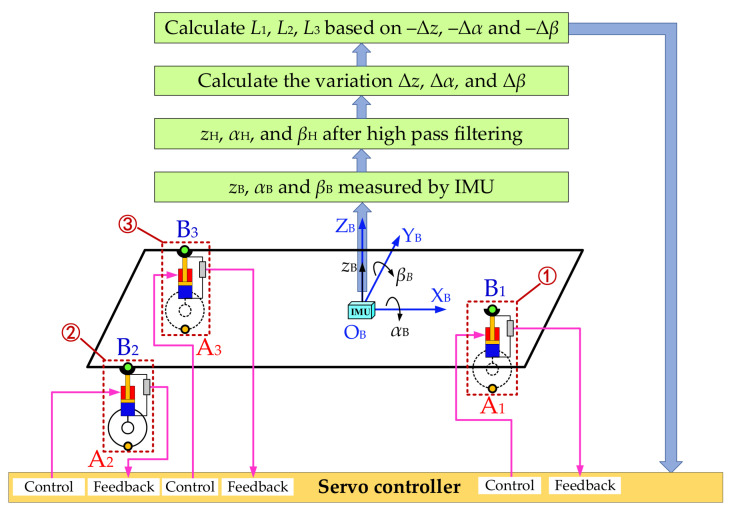
Schematic diagram of the principle of the control strategy.

According to the fulcrum equivalence principle described above, for the multi-axle vehicle shown in Figure 2, all suspension units can be divided into three groups. All of the suspension units of the first m axles can be listed as the first group, all left suspension units of the last n axles can be listed as the second group and all right suspension units of the last n axles can be listed as the third group. Connect the rodless cavity and rod cavity of all suspension servo actuating cylinders in each group in turn, then the 2 (m + n) support points of the suspension servo actuator cylinder on the vehicle body can be equivalent to three support points: B_1_, B_2_ and B_3_, and the 2 (m + n) grounding points between tire and ground can be equivalent to three grounding points: A_1_, A_2_ and A_3_. Regarding the triangle B_1_B_2_B_3_ as the moving platform, the triangleA_1_A_2_A_3_ as the base platform, and the three line segments A_1_B_1_, A_2_B_2_ and A_3_B_3_ as the drive rods, the whole vehicle can be regarded as being equivalent to a 3-DOF parallel mechanism like the 3-RPS configuration, as shown in Figure 4. Based on this equivalent transformation, the three groups of suspension units in Figure 4 can be equivalently replaced by the three equivalent suspension units (Suspension unit No.①, suspension unit No.② and suspension unit No.③) in Figure 5. The control of the three groups of suspension servo cylinders can be equivalent to the control of the three equivalent suspension units: ①, ② and ③.

On the basis of the above analysis and the equivalent transformation, an ASCS based on IMU for multi-axle emergency rescue vehicles can be proposed. The specific principle of the control strategy will be explained as follows in combination with Figure 5.

Establish a rectangular coordinate system O_B_-X_B_Y_B_Z_B_ with the center O_B_ of the IMU as the origin, as shown in Figure 5. Take the forward direction of the vehicle as the X_B_-axis’ direction, and take the upward direction perpendicular to the plane X_B_O_B_Y_B_ as the Z_B_-axis’ direction. The direction of the Y_B_-axis is determined by the right-hand rule. The vertical displacement at the coordinate origin O_B_ measured by the IMU is *z*_B_, the roll angle is *α*_B_ and the pitch angle is *β_B_*.

At the moment of *t* (the present moment), the vertical displacement at the origin O_B_ measured by the IMU is *z_B-t_*, the roll angle is *α_B-t_*, and the pitch angle is *β_B-t_*. Then, implement a high-pass filter with a cutoff frequency of *ω* on *z_B-t_*, *α_B-t_*, and *β_B-t_*, and obtain the filtered vertical displacement as *z_H-t_*, the roll angle as *α_H-t_*, and the pitch angle as *β_H-t_*. The function of the high-pass filter is to remove the parts of the vertical displacement, roll angle and pitch angle that change slowly when the vehicle is driving on a gentle slope. After filtering, the suspension servo actuator will not have the problem of travel saturation due to the vehicle driving on a slope, and the vehicle can travel along the envelope surface of the gentle slope.

At the moment of (*t*–1) (the moment before *t*), the vertical displacement was *z_H-t-_*_1_, the roll angle was *α_H-t-_*_1_ and the pitch angle was *β_H-t_*_-1_ after high-pass filtering. Then, the value of the vertical displacement variation, the roll angle variation and the pitch angle variation at the moment of *t* relative to the moment of (*t*–1) can be obtained by the equation (1):

(1) $$\left\{\begin{array}{l} \Delta z=z_{H-t}-z_{H-t-1}\\ \Delta \alpha =\alpha_{H-t}-\alpha_{H-t-1}\\ \Delta \beta =\beta_{H-t}-\beta_{H-t-1} \end{array}\right.$$

Take –Δ*z*, –Δ*α* and –Δ*β* as the moving platform attitude corrections of the multi-axle vehicle equivalent 3-DOF parallel mechanism, and calculate the displacement *L*_1_, *L*_2_ and *L*_3_ of the three equivalent suspension servo cylinders by finding the inverse solution of the position of the parallel mechanism. Then, the servo controller performs closed-loop control of the three equivalent suspension servo cylinders based on the calculated target displacements *L*_1_, *L*_2_ and *L*_3_ with the signal feedback of the displacement sensor, so as to realize the effective compensation and control of the body attitude of the multi-axle emergency rescue vehicle.

In the actual control process, the control of *L*_1_, *L*_2_ and *L*_3_ were realized by controlling the average displacement of all suspension servo actuating cylinders in each group. Take the second group shown in Figure 4 as an example, and the rest are the same. The number of the actuators of the second group is *n*. Therefore, let *x*_1_, *x*_2_… *x*_n_ be the actuators’ displacement value. When the actuator piston rods are in the mid-stroke position, the value of *x*_1_, *x*_2_… *x*_n_ are 0. When the piston rods are extended, the value of *x*_1_, *x*_2_… *x*_n_ are positive, and when the piston rods are shortened, the value of *x*_1_, *x*_2_… *x*_n_ are negative. The average displacement of all the actuators in the group is *x*= (*x*_1_+*x*_2_+…+ *x*_n_)/n, and the actual control object is *x*. Therefore, the control of *L*_1_, *L*_2_ and *L*_3_ were the control of *x* of each group.

## 3. Application Example of the ASCS Based on IMU

This section will combine the project subject and take the three-axle emergency rescue vehicle as the research object in order to study the ASCS proposed in this paper.

### 3.1. Construction of the Equivalent 3-DOF Parallel Mechanism

**Figure 6:**
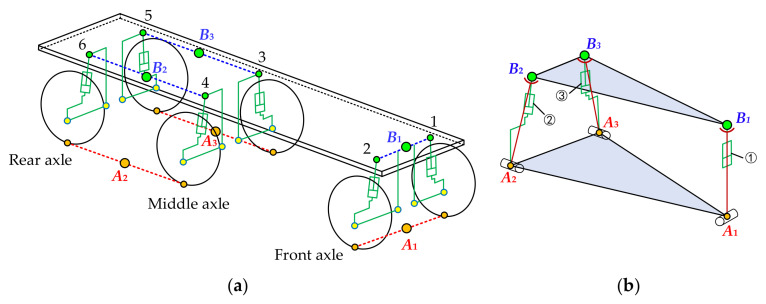
Construction of equivalent 3-DOF parallel mechanism for three-axle vehicle: (**a**) Grouping and interconnection of the suspension units; (**b**) equivalent 3-DOF parallel mechanism.

According to the suspension unit grouping method and the suspension servo actuator cylinder interconnection method shown in Figure 4, the six suspension units shown in Figure 6a are divided into three groups. The two suspension units on the left and right sides of the front axle are the first group, the two suspension units on the right side of the middle and rear axle are the second group, and the two suspension units on the left side of the middle and rear axle are the third group. Connect all rodless cavities and all rod cavities of the suspension servo actuator cylinders in each group. Then, the points B_1_, B_2_ and B_3_ are the equivalent support points of point 1 and point 2, point 4 and point 6, and point 3 and point 5, respectively. B_1_, B_2_ and B_3_ are respectively located at the midpoint of the connection between the two points in each group. Corresponding to B_1_, B_2_ and B_3_, A_1_ A_2_ and A_3_ are the equivalent grounding points of each group tire grounding points.

On the basis of the equivalent support points and the equivalent tire grounding points, the equivalent 3-DOF parallel mechanism can be constructed as shown in Figure 6b. In Figure 6b, ①, ②, and ③ are the equivalent suspension servo cylinders, and their displacements are *l*_1_, *l*_2_ and *l*_3_, respectively. *L*_1_, *L*_2_ and *L*_3_ are the lengths of the driving rods A_1_B_1_, A_2_B_2_ and A_3_B_3_, respectively.

### 3.2. Inverse Position Solution of the Equivalent 3-DOF Parallel Mechanism

As can be seen from Figure 6b, compared with the classical 3-RPS, the equivalent 3-DOF parallel mechanism constructed in this paper has certain particularities. Firstly, due to the vehicle body structure, triangleB_1_B_2_B_3_ is not an equilateral triangle but an isosceles triangle, which makes it impossible to directly quote the ready-made position inverse solution formula of 3-RPS parallel mechanism, and it needs to be deduced again. Secondly, under the influence of suspension geometry, when the tire jumps vertically, the track will change, leading to a change in the size and shape of the triangleA_1_A_2_A_3_. Therefore, it is necessary to analyze the change of the vehicle track. Thirdly, in Figure 6b, it can be seen that neither of the drive rods A_2_B_2_ and A_3_B_3_ are on the same axis as the equivalent suspension servo cylinders ② and ③. After calculating the length of the drive rod, it is necessary to calculate the length of the equivalent suspension servo cylinder according to the geometric relationship of the suspension.

Considering the above problems, if the position inverse solution of the equivalent 3-DOF parallel mechanism is required to be solved, the kinematics of the suspension needs to be analyzed.

#### 3.2.1. Kinematic Analysis of Single Wishbone Suspension

**Figure 7:**
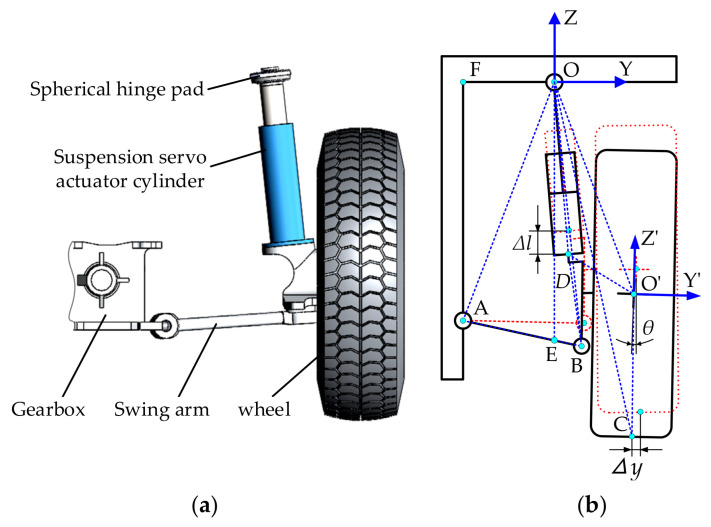
Single wishbone suspension structure of 1/6 vehicle: (**a**) 3D drawing; (**b**) 2D structure diagram.

The suspension of the three-axle emergency rescue vehicle studied in this paper is a single wishbone independent suspension, as shown in Figure 7a. The piston rod of suspension servo actuator cylinder is hinged with the vehicle frame through spherical hinge pad, and the cylinder barrel is rigidly connected with the wheel edge; one end of the swing arm is hinged with the gearbox shell, and the other end is hinged with the wheel edge.

Figure 7b shows a plan view of the suspension structure. In Figure 7b, the red dashed line indicates the balanced position of the suspension, and at this time, the piston rod of the suspension cylinder is in the middle position. The black solid line indicates the state of the suspension movement to a certain moment. Point O is the hinge point between the suspension cylinder piston rod and the vehicle frame; point O’ is the wheel rotation center; point A is the hinge point between gearbox shell and swing arm; point B is the hinge point between wheel edge and swing arm; point C is the tire grounding point; point D is the center of the cylinder base; the line OE direction is vertical, and its intersection with the line AB is point E. The camber angle is *θ*, and its value is 0° when the piston rod of suspension cylinder is in the middle position. Establish the rectangular coordinate system OXYZ with point O as the origin and establish the rectangular coordinate system O′X′Y′Z′ with point O′ as the origin. Both the OX axis and the O′X′ axis point in the forward direction of the vehicle. The displacement change of suspension servo actuator cylinder is Δ*l*; the change of grounding point C in Y-axis is Δ*y*. The rest of the geometric parameters in Figure 7b are shown in Table 1.

From the geometric relationship shown in Figure 7b, the following equation can be obtained according to the trigonometric function:

(2) $$l_{OB}^{2}=l_{OD}^{2}+l_{BD}^{2}-2l_{OD}l_{BD}\cos\alpha_{1}$$

(3) $$l_{AB}^{2}=l_{OA}^{2}+l_{OB}^{2}-2l_{OA}^{}l_{OB}^{}\cos\alpha_{2}$$

(4) $$l_{BD}^{2}=l_{OD}^{2}+l_{OB}^{2}-2l_{OD}^{}l_{OB}^{}\cos\alpha_{3}$$

(5) $$l_{OO^{’}}^{2}=l_{OD}^{2}+l_{O^{’}D}^{2}-2l_{OD}^{}l_{O^{’}D}^{}\cos\alpha_{5}$$

(6) $$l_{O^{’}D}^{2}=l_{OD}^{2}+l_{OO^{’}}^{2}-2l_{OD}^{}l_{OO^{’}}^{}\cos\alpha_{7}$$

The simultaneous Equations (2)–(6), the α_2_, α_3_ and α_7_ can be expressed as

(7) $$\alpha_{2}=\arccos\left(\frac{l_{OA}^{2}+l_{OD}^{2}+l_{BD}^{2}-l_{AB}^{2}-2l_{OD}^{}l_{BD}^{}\cos\alpha_{1}}{2l_{OA}^{}\sqrt{l_{OD}^{2}+l_{BD}^{2}-2l_{OD}^{}l_{BD}^{}\cos\alpha_{1}}}\right)$$

(8) $$\alpha_{3}=\arccos\left(\frac{l_{OD}^{}-l_{BD}^{}\cos\alpha_{1}}{\sqrt{l_{OD}^{2}+l_{BD}^{2}-2l_{OD}^{}l_{BD}^{}\cos\alpha_{1}}}\right)$$

(9) $$\alpha_{7}=\arccos\left(\frac{l_{OD}^{}-l_{O^{’}D}^{}\cos\alpha_{5}}{\sqrt{l_{OD}^{2}+l_{O^{’}D}^{2}-2l_{OD}^{}l_{O^{’}D}^{}\cos\alpha_{5}}}\right)$$

Let the coordinate of the wheel rotation center O′ in the coordinate system OXYZ be (xo’o,yo’o,zo’o), and its coordinate value can be obtained by the following equation

(10) xo’o=0yo’o=lOO’sinα6+α7zo’o=−lOO’cosα6+α7

where $\alpha_{6}=\alpha_{2}-\alpha_{3}-\alpha_{4}$, and $\;\alpha_{4}=\arcsin\left(l_{OF}/l_{OA}\right)$.

Let the coordinates of the wheel grounding point C in the coordinate systems O′X′Y′Z′ and OXYZ be (xco’,yco’,zco’) and (xco,yco,zco), respectively, where

(11) xco’=0yco’=0zco’=−lO’C 

According to the displacement matrix method [29], the following equation can be obtained

(12) xcoycozco=Rxco’yco’zco’+xo’oyo’ozo’o

where R is rotation matrix and its value is

(13) $$\left[\begin{array}{ccc} 1 & 0 & 0\\ 0 & \cos\theta & -\sin\theta \\ 0 & \sin\theta & \cos\theta \end{array}\right]$$

Substituting Equations (7)–(11) and (13) into Equation (12), the (xco,yco,zco) can be expressed as

(14)  xcoycozco=0lO’Csinθ+lOD2+lO’D2−2lODlO’Dcosα5sinα6+α7−lO’Ccosθ−lOD2+lO’D2−2lODlO’Dcosα5cosα6+α7

It can be seen from Figure 7b that the displacement change of suspension servo actuator cylinder Δ*l* can be expressed as

(15) $$\Delta l=l_{OD}-l_{OD}{}_{{}_{0}}$$

where $l_{OD}{}_{{}_{0}}$ is the length of $l_{OD}$ when the piston rod of suspension cylinder is in the middle position.

Substitute Equation (15) into Equation (14), and Equation (14) can be expressed as

(16)  xcoycozco=0lO’Csinθ+lOD0+Δl2+lO’D2−2lOD0+ΔllO’Dcosα5sinα6+α7−lO’Ccosθ−lOD0+Δl2+lO’D2−2lOD0+ΔllO’Dcosα5cosα6+α7

According to Equation (16), the change of the grounding point C in Y-axis can be expressed respectively by the following equation

(17) Δy=yco−yc0o

where yc0o is the value of yco when the Δl is 0.

As can be seen from Figure 7b, the length of the line segment OC is the length of the driving rods A_2_B_2_ and A_3_B_3_. According to Equation (16), the length of the line segment OC can be expressed as

(18) lOC=yc2o+zc2o

The change of the line segment OC is expressed as

(19) ΔlOC=yc2o+zc2o−yc02o+zc02o

**Figure 8:**
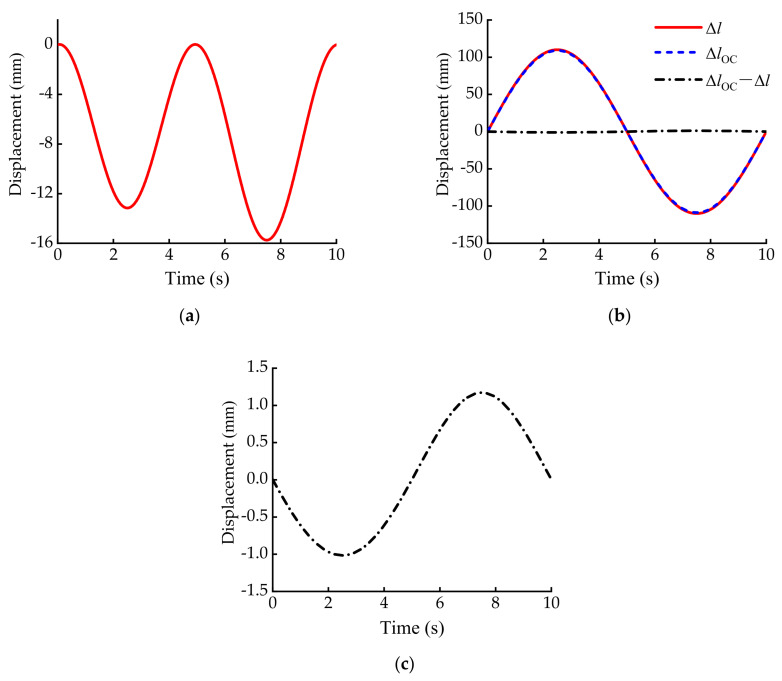
The curve of suspension geometric parameters with time: (**a**) the curve of Δ*y* with time *t*; (**b**) the comparative curve of Δ*l*, Δ*l_OC_* and (Δ*l_OC_*–Δ*l*) with time *t*; (**c**) the enlarged curve of the (Δ*l_OC_*–Δ*l*).

The stroke of the suspension servo cylinder of the three-axis emergency rescue vehicle studied in this paper is − 110~+110 mm. That means the maximum variation range of Δ*l* is−110~+110 mm. Assuming that the vehicle frame is fixed, make the suspension servo actuator cylinder act according to the signal Δ*l* = 110 sin(0.2*πt*) mm. Then, the curves of Δ*y*, Δ*l*, Δ*l_OC_* and the (Δ*l_OC_*–Δ*l*) with time *t* can be obtained as shown in Figure 8.

As can be seen from Figure 8a, in a motion cycle of the suspension servo actuator cylinder, the maximum value of Δ*y* is only 15.75 mm, which can be ignored compared with the 2550 mm track of the whole vehicle when all of the suspension servo actuators are in the middle position. For the convenience of engineering application and calculation, we assume that the grounding point is fixed relative to the base platform.

As can be seen from Figure 8b,c, in a motion cycle of the suspension servo actuator cylinder, the curve Δ*l* and the curve Δ*l_OC_* are almost coincident. The maximum value of their difference is only 1.17 mm. For the drive rod A_2_B_2_ and the equivalent suspension servo cylinder ②, although they are not on the same axis, the displacement change between the A_2_B_2_ and ② is very small. Therefore, the length *L*_2_ of the drive rod A_2_B_2_ can be used as the control displacement of the equivalent suspension servo cylinder ②. This method is also applicable to drive rod A_3_B_3_ and suspension servo cylinder ③.

On the basis of the above analysis, the position inverse solution of the equivalent 3-DOF parallel mechanism shown in Figure 6b will be obtained next.

#### 3.2.2. Solve the Inverse Solution

**Figure 9:**
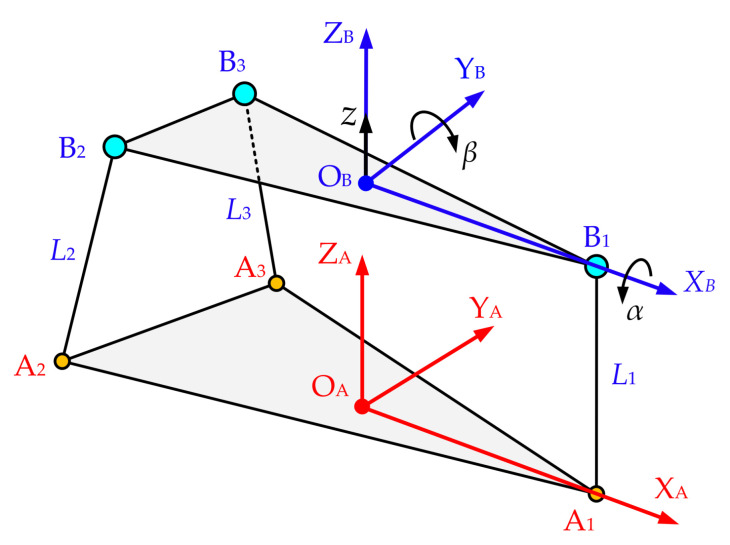
Equivalent 3-DOF parallel mechanism and the coordinate system.

Establish the rectangular coordinate system O_A_-X_A_Y_A_Z_A_ with the outer center O_A_ of the base platform as the original center and the rectangular coordinate system O_B_-X_B_Y_B_Z_B_ with the outer center O_B_ of the moving platform as the original center, as shown in Figure 9. The Z_A_-axis is perpendicular to the base platform and is pointing upwards. The X_A_-axis passes through point A_1_ and is along the direction of O_A_X_A_. The direction of Y_A_-axis is determined by the right-hand rule. The Z_B_-axis is perpendicular to the moving platform and points directly above. The X_B_-axis passes through point B_1_ and is along the direction of O_B_X_B_. The direction of the Y_B_-axis is determined by the right-hand rule. The coordinate system O_B_-X_B_Y_B_Z_B_ is fixedly connected with the moving platform. *α* and *β* are the roll angle and pitch angle of the moving platform, respectively. The *z* is the vertical displacement of the moving platform.

Based on the establishment of the above coordinate system, the coordinate values of B_1_, B_2_ and B_3_ in the coordinate system O_B_-X_B_Y_B_Z_B_ are

(20) PB1OB=xB1OByB1OB0,PB2OB=xB2OByB2OB0,PB3OB=xB3OByB3OB0

The coordinate values of A_1_, A_2_ and A_3_ in the coordinate system O_A_-X_A_Y_A_Z_A_ are

(21) PA1OA=xA1OAyA1OA0,PA2OA=xA2OAyA2OA0,PA3OA=xA3OAyA3OA0

The homogeneous transformation matrix of coordinate system O_B_-X_B_Y_B_Z_B_ relative to coordinate system O_A_-X_A_Y_A_Z_A_ is

(22) T=RPOBOA01

where ***R*** is the posture rotation matrix of coordinate system O_B_-X_B_Y_B_Z_B_ relative to coordinate system O_A_-X_A_Y_A_Z_A_. The vector POBOA is the position vector of the origin O_B_ in the coordinate system O_A_-X_A_Y_A_Z_A_. The expressions of matrix ***R*** and vector POBOA are

(23) $$R=R_{Z}\left(\gamma \right)R_{Y}\left(\beta \right)R_{X}\left(\alpha \right)$$

(24) POBOA=xOBOAyOBOAzOBOA

where

(25) $$R_{X}\left(\alpha \right)=\left[\begin{array}{ccc} 1 & 0 & 0\\ 0 & c\alpha & -s\alpha \\ 0 & s\alpha & c\alpha \end{array}\right],R_{Y}\left(\beta \right)=\left[\begin{array}{ccc} c\beta & 0 & s\beta \\ 0 & 1 & 0\\ -s\beta & 0 & c\beta \end{array}\right],R_{Z}\left(\gamma \right)=\left[\begin{array}{ccc} c\gamma & -s\gamma & 0\\ s\gamma & c\gamma & 0\\ 0 & 0 & 1 \end{array}\right]$$

The coordinate values of B_1_, B_2_ and B_3_ in the coordinate system O_A_-X_A_Y_A_Z_A_ can be expressed as

(26) PBiOA1=TPBiOB1i=1,2,3

Substitute Equations (20)–(25) into Equation (26), the PBiOAi=1,2,3 can be expressed as

(27) PBiOA= xOBOA−yBiOBcαsγ−sαsβcγ+xBiOBcβcγyOBOA+yBiOBcαcγ+sαsβsγ+xBiOBcβsγzOBOA−xBiOBsβ+yBiOBcβsαi=1,2,3

In Figure 9, due to the special arrangement of revolute pairs, B_1_ can only move in the plane yOA=0, and B_2_, B_3_ can only move in the plane xA2OA=xA3OA=xOA. Therefore, the following equation can be obtained:

(28) xOBOA−yB2OBcαsγ−sαsβcγ+xB2OBcβcγ=xOAxOBOA−yB3OBcαsγ−sαsβcγ+xB3OBcβcγ=xOAyOBOA+yB1OBcαcγ+sαsβsγ+xB1OBcβsγ=0

In coordinate system O_B_-X_B_Y_B_Z_B_, the *x* coordinate and *y* coordinate of B_1_, B_2_ and B_3_ have the following relationship

(29) yB1OB=0xB2OB=xB3OB=xOByB2OB=−yB3OB=yOB

Solving the system of Equation (28), we can obtain the following equations

(30) xOBOA=xOA−xcβcγOB

(31) yOBOA=−xB1OBcβsγ−yB1OBcαcγ+sαsβsγ

(32) $$\left\{\begin{array}{l} s\gamma =\frac{s\alpha s\beta }{\sqrt{s^{2}\alpha s^{2}\beta +c^{2}\alpha }}\\ c\gamma =\frac{c\alpha }{\sqrt{s^{2}\alpha s^{2}\beta +c^{2}\alpha }} \end{array}\right.$$

Substituting Equations (29)–(32) into Equation (27), the PBiOAi=1,2,3 can be expressed as

(33) PB1OA= xOA+xB1OB−xOBcαcβs2αs2β+c2α0zOBOA−xB1OBsβ

(34) PB2OA= xOAyOBs2αs2β+c2α+xOB−xB1OBsαsβcβs2αs2β+c2αzOBOA−xsβOB+ycβsαOB

(35) PB3OA= xOA−yOBs2αs2β+c2α+xOB−xB1OBsαsβcβs2αs2β+c2αzOBOA−xsβOB−ycβsαOB

Based on PAiOAi=1,2,3 and PBiOAi=1,2,3, the length $L_{i}\left(i=1,2,3\right)$ of the drive rod can be expressed as

(36) L1=xAiOA−xOA+xOB−xBiOBcαcβs2αs2β+c2α2+yAiOA2+xB1OBsβ−zOBOA2

(37) L2=xA2OA−xOA2+yA2OA−yOBs2αs2β+c2α−xOB−xB1OBsαsβcβs2αs2β+c2α2+xsβOB−zOBOA−ycβsαOB2

(38) L3=xA3OA−xOA2+yA3OA+yOBs2αs2β+c2α−xOB−xB1OBsαsβcβs2αs2β+c2α2+xsβOB−zOBOA+ycβsαOB2

As can be seen from Figure 9, the *x* coordinate and *y* coordinate of A_1_, A_2_ and A_3_ have the following relationship

(39) yA1OA=0xA2OA=xA3OA=xOAyA2OA=−yA3OA=yOA

Replace zOBOA, *α* and *β* of Equations (36)–(38) with zOB0OA−Δz, −Δα and −Δβ, respectively, and according to Equation (39), Equations (36)–(38) can be further simplified as

(40) L1=xA1OA−xOA+xOB−xB1OBcΔαcΔβs2Δαs2Δβ+c2Δα2+Δz−xB1OBsΔβ−zOB0OA2

(41) L2=yOA−yOBs2Δαs2Δβ+c2Δα−xOB−xB1OBsΔαsΔβcΔβs2Δαs2Δβ+c2Δα2+Δz−xsΔβOB+ycΔβsΔαOB−zOB0OA2

(42) L3=−yOA+yOBs2Δαs2Δβ+c2Δα−xOB−xB1OBsΔαsΔβcΔβs2Δαs2Δβ+c2Δα2+Δz−xsΔβOB−ycΔβsΔαOB−zOB0OA2

where zOB0OA is the Z_A_-axis coordinate value of O_B_ in the coordinate system O_A_-X_A_Y_A_Z_A_ when all suspension servo cylinders are in the middle position.

So far, the derivation of the inverse position solution of the equivalent 3-DOF parallel mechanism has been completed. When Δz,Δα and Δβ are known, the variation of the driving rod can be obtained through Equations (40)–(42), and then the displacement control variation of each equivalent suspension servo actuator cylinder can be obtained.

### 3.3. Design High-Pass Filter

According to the control principle described in Section 2, it is necessary to design a high-pass filter. For the convenience of subsequent engineering application, we will design the high-pass filter based on the principle of first-order RC high-pass filter circuit [26].

**Figure 10:**
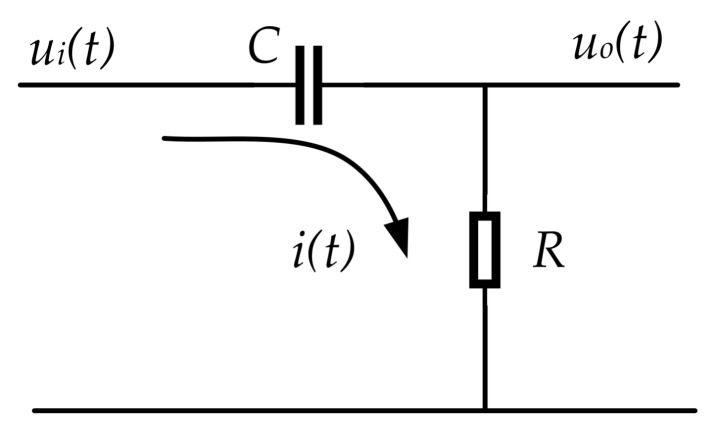
Schematic diagram of the first-order RC high-pass filter circuit.

Figure 10 shows the first-order RC high-pass filter circuit. *u_i_(t)* is the input voltage. *u_o_(t)* is the output voltage. *i(t)* is the current. *R* is resistance. *C* is the capacitance.

According to the Kirchhoff’s Law and Ohm’s Law, we obtain the following equation:

(43) $$\left\{\begin{array}{l} u_{i}\left(t\right)=\frac{1}{C}\int i\left(t\right)dt+i\left(t\right)R\\ u_{o}\left(t\right)=i\left(t\right)R \end{array}\right.$$

Implementing the Laplace transform on Equation (43), the transfer function between *u_o_(t)* and *u_i_(t)* can be expressed as

(44) $$G\left(s\right)=\frac{U_{o}\left(s\right)}{U_{i}\left(s\right)}=\frac{RCs}{RCs+1}$$

By converting Equation (44) into frequency characteristic function, we obtain

(45) $$\left\{\begin{array}{l} \left|G\left(j\omega \right)\right|=\frac{\omega RC}{\sqrt{1+{\left(\omega RC\right)}^{2}}}\\ \varphi \left(j\omega \right)=\arctan\frac{1}{\omega RC} \end{array}\right.$$

As can be seen from Equation (45), when ω=0,$\left|G\left(j\omega \right)\right|=0$; when $\omega =\frac{1}{RC}$, $\left|G\left(j\omega \right)\right|=\frac{\sqrt{2}}{2}$; when ω→+∞,$\left|G\left(j\omega \right)\right|=1$. Therefore, it can be seen that the RC circuit shown in Figure 10 has the characteristic of filtering low-frequency voltage signals and retaining high-frequency voltage signals. Based on this principle, the transfer function of the high-pass filter can be designed as shown in the following equation

(46) $$H\left(j\omega \right)=\frac{1}{1-j\frac{\omega_{c}}{\omega }}$$

where $\omega_{c}$ is the cutoff angle frequency, and $\omega_{c}=2\pi f_{c}$. Where $f_{c}$ is cutoff frequency.

The value of $f_{c}$ is determined by combining the following two methods:

1. When the vehicle is stationary on a horizontal road, the determined cutoff frequency $f_{c1}$ must ensure that the output body pose after high-pass filtering converges to 0;
2. When the vehicle is stationary on the maximum allowable slope, the determined cutoff frequency $f_{c2}$ must ensure that the output body pose after high-pass filtering converges to a small value, which must be within the allowable error range of suspension system stability control;
3. Take the smaller value between $f_{c1}$ and $f_{c2}$.

## 4. Actual Vehicle Experiment

**Figure 11:**
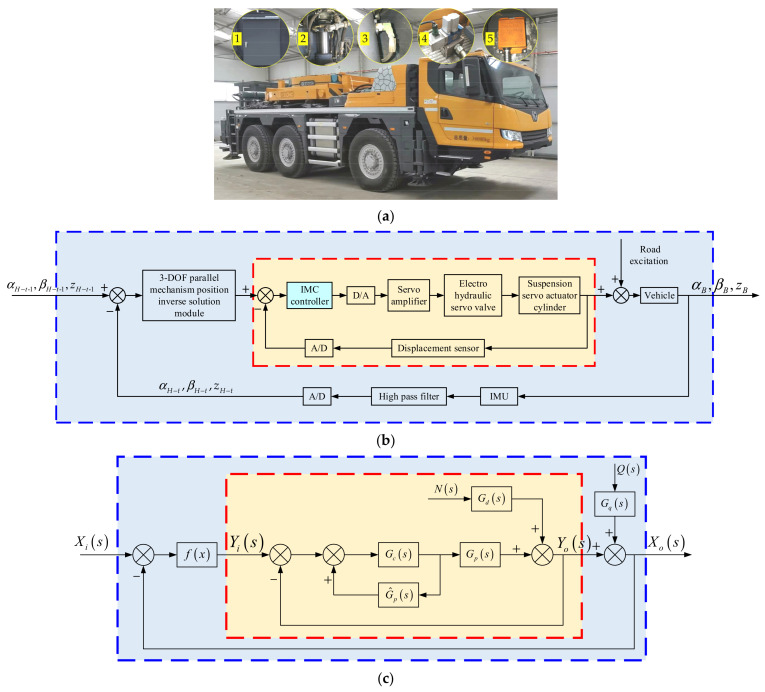
Vehicle experiment platform and schematic diagram of active suspension control system: (**a**) Vehicle experiment platform: (1) Electric control system. (2) Suspension servo actuator cylinder. (3) Displacement sensor. (4) Servo valve. (5) IMU; (**b**) Schematic diagram of active suspension control system; (**c**) Block diagram of active suspension control system.

To verify the effect of the proposed control strategy in actual engineering applications, we built a three-axle six-wheel vehicle suspension system experiment platform, as shown in Figure 11a. The experiment platform integrates two suspension systems: the active suspension system which uses the ASCS proposed in this paper and the hydro-pneumatic suspension system. The active suspension and the hydro-pneumatic suspension can be mutually switched through the opening and closing of related valve groups. The active suspension system mainly includes IMU, hydraulic system, control system, suspension servo actuator cylinder, and displacement sensor corresponding to the actuator cylinder.

### 4.1. Stability Analysis of Active Suspension Control System

The control system of the active suspension is shown in Figure 11b, and its block diagram is shown in Figure 11c. This control system is a double closed loop control system. The inner feedback loop (the part surrounded by the red wireframe) is the position control loop of the actuator and the outer feedback loop (the part surrounded by the blue wireframe) is the vehicle attitude control loop. The control strategy used in the inner feedback loop is Internal Model Control (IMC) [30].

In Figure 11c, $X_{i}\left(s\right)$ and $X_{o}\left(s\right)$ are the initial attitude and the attitude measured by the sensors, respectively. $Y_{i}\left(s\right)$ is the displacement calculated by 3-DOF parallel mechanism position inverse solution module and $Y_{o}\left(s\right)$ is the displacement measured by the displacement sensor. The 3-DOF parallel mechanism position inverse solution module is equivalent to a constant *K* and its max value is 0.22 because of the stroke of the actuator. $G_{p}\left(s\right)$ is the controlled object, $G_{c}\left(s\right)$ is the IMC controller and ${\hat{G}}_{p}\left(s\right)$ is the internal model. $G_{d}\left(s\right)$ is the transfer function of the actuator load interference channel and $G_{q}\left(s\right)$ is the transfer function of the road interference channel.

The $X_{o}\left(s\right)$ can be expressed by the following equation:

(47) $$\begin{array}{l} X_{o}\left(s\right)=\frac{KG_{c}\left(s\right)G_{p}\left(s\right)}{1+G_{c}\left(s\right)\left[G_{p}\left(s\right)-{\hat{G}}_{p}\left(s\right)\right]+KG_{c}\left(s\right)G_{p}\left(s\right)}X_{i}\left(s\right)\\ +\frac{\left[1-G_{c}\left(s\right){\hat{G}}_{p}\left(s\right)\right]G_{d}\left(s\right)}{1+G_{c}\left(s\right)\left[G_{p}\left(s\right)-{\hat{G}}_{p}\left(s\right)\right]+KG_{c}\left(s\right)G_{p}\left(s\right)}N\left(s\right)\\ +\frac{\left\{1+G_{c}\left(s\right)\left[G_{p}\left(s\right)-{\hat{G}}_{p}\left(s\right)\right]\right\}G_{q}\left(s\right)}{1+G_{c}\left(s\right)\left[G_{p}\left(s\right)-{\hat{G}}_{p}\left(s\right)\right]+KG_{c}\left(s\right)G_{p}\left(s\right)}Q\left(s\right) \end{array}$$

where

(48) $$G_{p}\left(s\right)=\frac{K_{o}}{s\left(\frac{s^{2}}{\omega_{h}^{2}}+\frac{2\zeta_{h}}{\omega_{h}}s+1\right)}$$

(49) $$G_{c}\left(s\right)=\frac{s\left(\frac{s^{2}}{\omega_{h}^{2}}+\frac{2\zeta_{h}}{\omega_{h}}s+1\right)}{K_{o}{\left(\lambda s+1\right)}^{3}}$$

The parameter $K_{o}$ is the open loop gain; $\omega_{h}$ is the natural frequency; $\zeta_{h}$ is the damping ratio; λ is the time constant, and it is the only parameter that needs to be adjusted of the IMC controller. The specific meaning or the derivation process of these parameters was described in a previously published article [30] and will not be repeated here.

During the design of the IMC controller, we chose $G_{p}\left(s\right)$ as the internal model [30], that is

(50) $${\hat{G}}_{p}\left(s\right)=G_{p}\left(s\right)$$

Substitute Equations (48)–(50) into Equation (47), and Equation (47) can be simplified to Equation (51)

(51) $$X_{o}\left(s\right)=\frac{K}{{\left(\lambda s+1\right)}^{3}+K}X_{i}\left(s\right)+\frac{G_{d}\left(s\right){\left(\lambda s+1\right)}^{3}-G_{d}\left(s\right)}{{\left(\lambda s+1\right)}^{3}+K}N\left(s\right)+\frac{{\left(\lambda s+1\right)}^{3}G_{q}\left(s\right)}{{\left(\lambda s+1\right)}^{3}+K}Q\left(s\right)$$

According to the basic criterion for judging the stability of linear systems, the sufficient and necessary condition for the stability of a system is that the roots of its characteristic equations are all in the left half plane of the *s* plane.

The characteristic equation of Equation (51) is

(52) $${\left(\lambda s+1\right)}^{3}+K=0$$

By solving Equation (52), its roots are

(53) $$s_{1}=-\frac{\sqrt[{3}]{K}+1}{\lambda }$$

(54) $$s_{2}=\frac{\sqrt[{3}]{K}-2}{2\lambda }+\frac{\sqrt{3}}{2}\frac{\sqrt[{3}]{K}}{\lambda }i$$

(55) $$s_{2}=\frac{\sqrt[{3}]{K}-2}{2\lambda }-\frac{\sqrt{3}}{2}\frac{\sqrt[{3}]{K}}{\lambda }i$$

Because λ > 0 and the max value of *K* is 0.22, the roots shown in Equations (53)–(55) are all in the left half plane of the s plane. Therefore, the active suspension control system is stable.

Based on the vehicle experiment platform, two groups of experiments will be carried out in this paper. The first group is a random road experiment and the second one is slope road experiment.

### 4.2. Random Road Experiment

#### 4.2.1. Experiment Scheme

**Figure 12:**
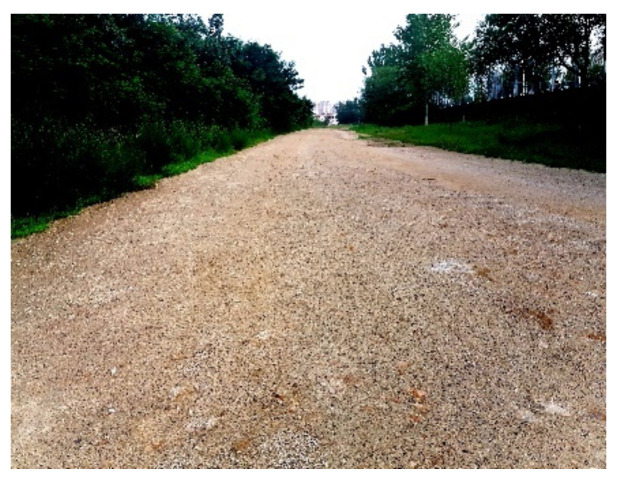
Gravel road.

The random road experiment uses the gravel road (Figure 12) as the experimental pavement, and uses the hydro-pneumatic suspension as the comparison.

During the experiment, first, start the control program of the active suspension system, drive the vehicle over a distance, and record the posture and acceleration changes of the vehicle body. Then, close the active suspension system program, switch to hydro-pneumatic suspension system, drive through the same road section at the same speed, and record the posture and acceleration changes of the vehicle body. The average speed during the experiment is 10 km/h.

#### 4.2.2. Experiment Result and Analysis

**Figure 13:**
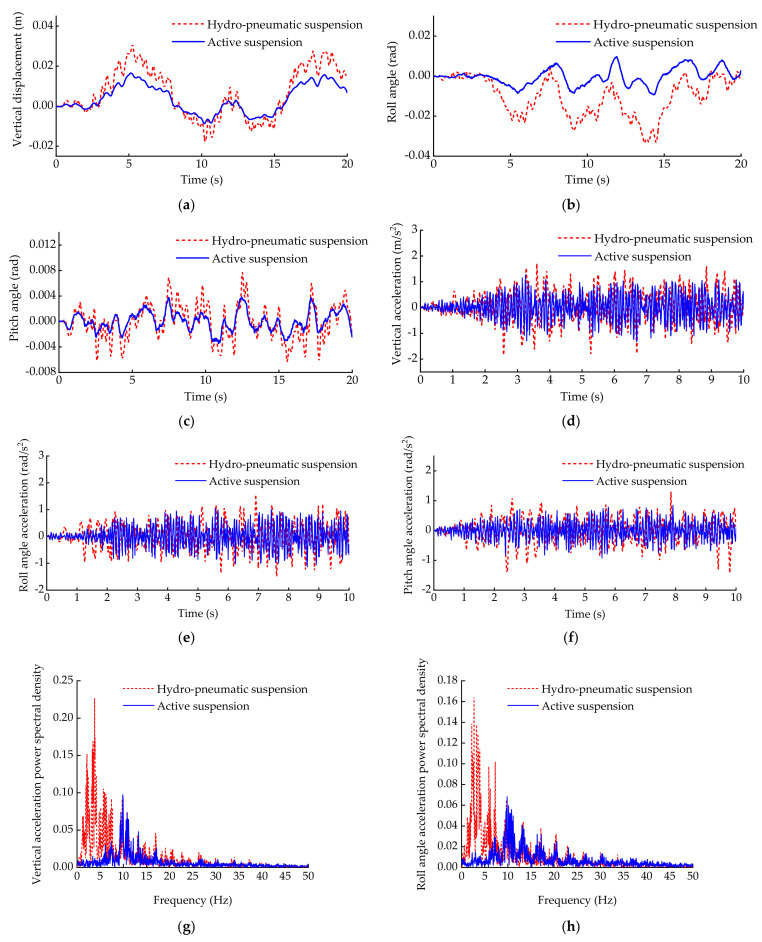

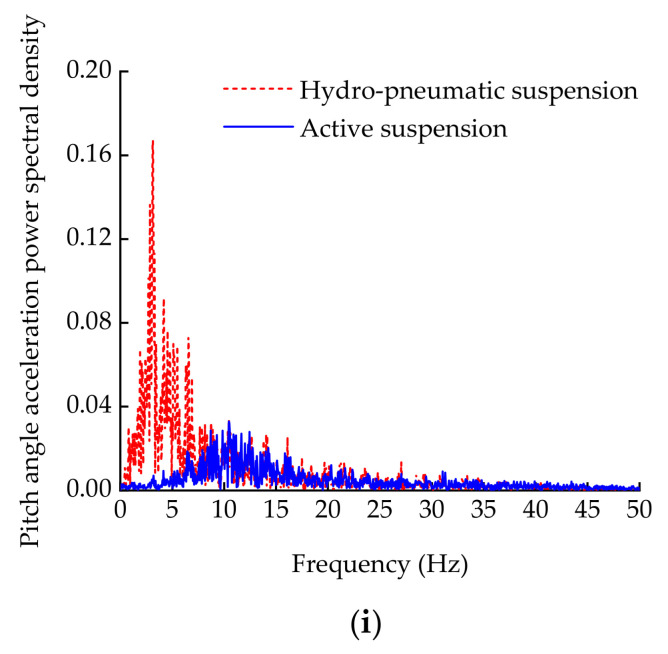
Attitude, acceleration of vehicle body and power spectral density of each acceleration: (**a**) vertical displacement of vehicle body; (**b**) roll angle of vehicle body; (**c**) pitch angle of vehicle body; (**d**) vertical acceleration of vehicle body; (**e**) roll angle acceleration of vehicle body; (**f**) pitch angle acceleration of vehicle body; (**g**) vertical acceleration power spectral density; (**h**) roll angle acceleration power spectral density; (**i**) pitch angle acceleration power spectral density.

**Figure 14:**
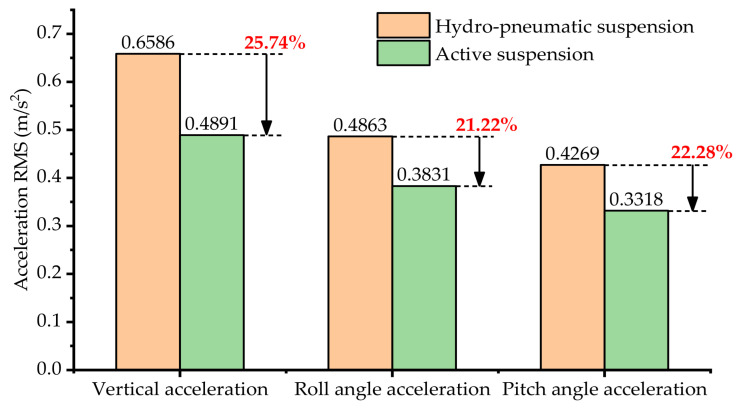
Comparison of acceleration RMS.

Figure 13a–c show a comparison of the vehicle body vertical displacement, roll angle and pitch angle. Figure 13d–f show a comparison of the vehicle body vertical acceleration, roll angle acceleration and pitch angle acceleration. Figure 13g–i show a comparison of the vehicle body vertical acceleration power spectral density, roll angle acceleration power spectral density and pitch angle acceleration power spectral density. Figure 14 shows a comparison of the acceleration root mean square (RMS) of the vertical acceleration, roll angle acceleration and pitch angle acceleration.

As can be seen from Figure 13a–c, the attitude fluctuation range of the vehicle body equipped with hydro-pneumatic suspension is large. Compared with the hydro-pneumatic suspension, the attitude fluctuation range of the vehicle body equipped with active suspension is smaller. Therefore, it can be seen that the active suspension which used the ASCS based on IMU proposed in this paper shows excellent control performance in stabilizing the body attitude.

As can be seen from Figure 13d–f, compared with hydro-pneumatic suspension, the ASCS based on IMU proposed in this paper can effectively attenuate the vehicle body vibration. Meanwhile, from Figure 13g–i, it can be seen that in the frequency range of less than 10 Hz, the amplitudes of the vertical acceleration, roll angle acceleration and pitch angle acceleration have been significantly reduced under the ASCS based on IMU proposed in this paper, while in the frequency range of 10–20 Hz or more, the amplitude of the active suspension was a little bigger than the hydro-pneumatic suspension. According to the acceleration RMS value shown in Figure 14, it can be seen that the vertical acceleration, roll angle acceleration and pitch angle acceleration of active suspension are reduced by 25.74%, 21.22% and 22.28%, respectively, compared with the hydro-pneumatic suspension. Therefore, the ASCS based on IMU proposed in this paper also has better ability with respect to improving the vehicle ride comfort.

On the basis of the above experiment results, it can be seen that compared with hydro-pneumatic suspension, the ASCS based on IMU proposed in this paper not only has the ability to stabilize the body attitude, but can also better improve the ride comfort of the vehicle.

### 4.3. Slope Road Experiment

#### 4.3.1. Experiment Scheme

We know from the description in Section 2 that the purpose of adding the high-pass filter is to suppress the displacement output saturation of the suspension servo cylinder when the vehicle is driving on a sloped road surface. To verify the effect of the high-pass filter in suppressing the saturation of displacement output, this section will conduct the slope road experiment.

The slope road surface experiment includes the longitudinal slope road experiment and the cross slope road experiment. The longitudinal slope road experiment is used to test the vertical movement and pitch motion of the vehicle body when the high-pass filter is enabled. The cross slope road experiment is used to test the roll motion of the vehicle body when the high-pass filter is enabled.

(1) Longitudinal slope road experiment scheme

**Figure 15:**
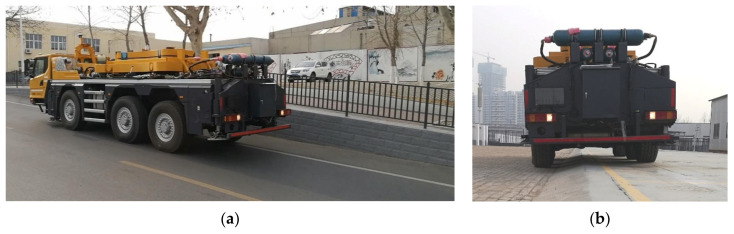
Slope road experiment: (**a**) longitudinal slope road experiment; (**b**) cross slope road experiment.

The longitudinal slope road experiment is divided into two groups. The first group of experiments only take the vertical displacement as the input to design the active suspension system program. In the second group of experiments, only the pitch angle is used as the input to design the active suspension system program. Both groups of experiments were carried out with the same experiment scheme. Start the active suspension program loaded with high-pass filter, drive the vehicle uphill, and record the changes of vehicle body attitude. Close the active suspension program loaded with high-pass filter and switch to the program without the high-pass filter loaded; go uphill at the same speed and path, and record the changes of vehicle body attitude. The experiment scenario is shown in Figure 15a.

(2) Cross slope road experiment scheme

Only take the roll angle as the input to design the active suspension system program. Start the active suspension program loaded with high-pass filter, drive the vehicle uphill, and record the changes of vehicle body attitude. Close the active suspension program loaded with high-pass filter and switch to the program without high-pass filter loaded, go uphill at the same speed and path, and record the changes of vehicle body attitude. The experiment scenario is shown in Figure 15b.

#### 4.3.2. Experiment Result and Analysis

Due to the longitudinal and cross slope roads being relatively flat and the vehicle running speed being low, the vehicle body vibration is small. Therefore, the vertical acceleration, pitch angle acceleration and roll angle acceleration of the vehicle body are not analyzed in the experiment results, but only the attitude of the vehicle body and the displacement of each equivalent suspension servo actuator cylinder are analyzed.

**Figure 16:**
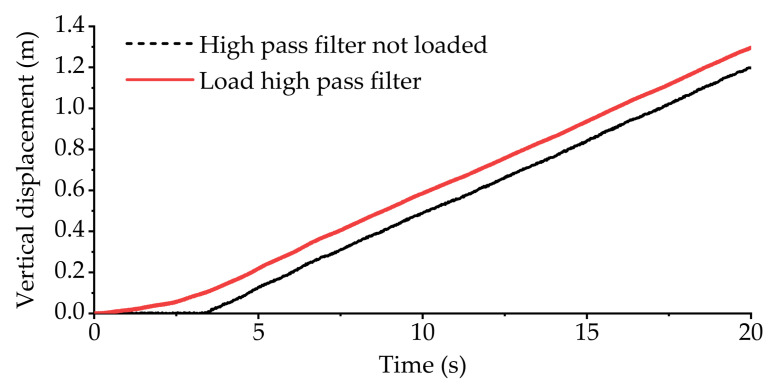
Vertical displacement of vehicle body in longitudinal slope experiment.

**Figure 17:**
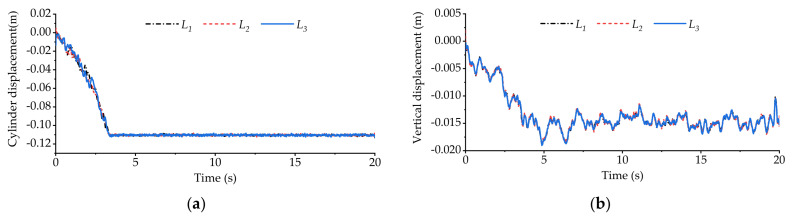
The displacement of equivalent suspension servo actuator cylinder when only vertical displacement is used as input in the longitudinal slope experiment: (**a**) displacement of equivalent suspension servo actuator cylinder without high-pass filter; (**b**) displacement of equivalent suspension servo actuator cylinder when high-pass filter is loaded.

Figure 16 shows the variation curve of vehicle body vertical displacement during longitudinal slope road experiment. Figure 17 shows the displacement variation curve of the equivalent suspension servo actuator cylinder when the high-pass filter is loaded and not loaded. As can be seen from Figure 16 and Figure 17a, when the active suspension system is not loaded with the high-pass filter, all the equivalent suspension servo actuator cylinders are extended to the longest position when the vehicle goes uphill for about 3.5 s, and they remain at the longest position during subsequent driving. However, from Figure 16 and Figure 17b, it can be seen that when the active suspension system is loaded with the high-pass filter, the displacement of the equivalent suspension servo actuator cylinder has been maintained between −0.01 m~−0.02 m after the vehicle goes uphill for about 5 s and has not reached the saturation limit position, indicating that the part with slow change frequency in the vertical displacement of the vehicle body has been basically filtered out.

**Figure 18:**
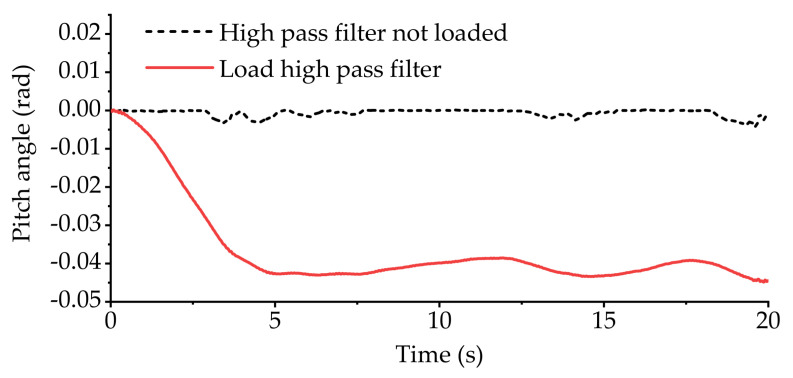
Pitch angle of vehicle body in longitudinal slope experiment.

**Figure 19:**
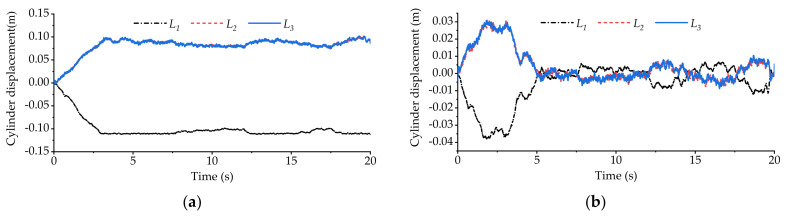
The displacement of equivalent suspension servo actuator cylinder when only pitch angle is used as input in the longitudinal slope experiment: (**a**) displacement of equivalent suspension servo actuator cylinder without high-pass filter; (**b**) displacement of equivalent suspension servo actuator cylinder when high-pass filter is loaded.

Figure 18 shows the variation curve of vehicle body pitch angle during longitudinal slope road experiment. Figure 19 shows the displacement variation curve of the equivalent suspension servo actuator cylinder when the high-pass filter is loaded and not loaded. It can be seen from Figure 18 that without the high-pass filter loaded, the pitch angle of the vehicle body fluctuates around 0 when the vehicle is running on the slope. The fluctuation range is small, and the vehicle body basically remains level. However, from Figure 19a, it can be seen that the front axle equivalent suspension servo actuator cylinder has been retracted to the shortest position, and it remains at the shortest position during subsequent driving. However, after loading the high-pass filter, the situation becomes different. It can be seen from Figure 18 and Figure 19b that each equivalent suspension servo cylinder first stretches and then contracts when the vehicle is driving uphill. After a period of time, the displacement of the equivalent suspension servo cylinder fluctuates around 0, which shows that the part with slow change frequency in the body pitch angle has been basically filtered out.

**Figure 20:**
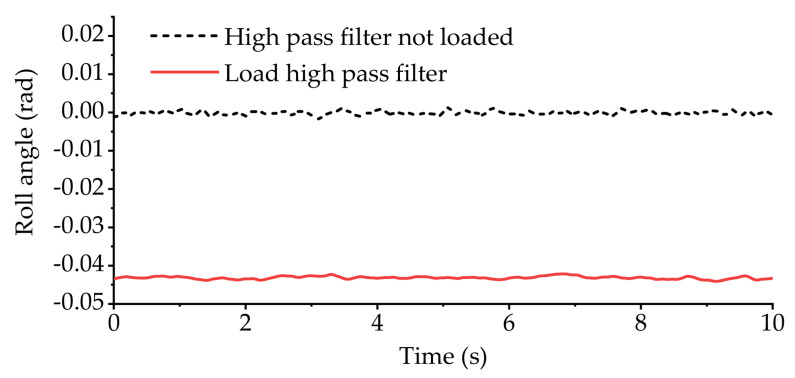
Roll angle of vehicle body in longitudinal slope experiment.

**Figure 21:**
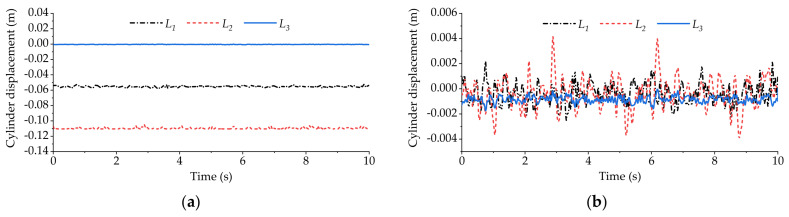
The displacement of equivalent suspension servo actuator cylinder in the cross slope experiment: (**a**) displacement of equivalent suspension servo actuator cylinder without high-pass filter; (**b**) displacement of equivalent suspension servo actuator cylinder when high-pass filter is loaded.

Figure 20 shows the variation curve of the vehicle body roll angle during the cross slope road experiment. Figure 21 shows the displacement variation curve of the equivalent suspension servo actuator cylinder when the high-pass filter is loaded and not loaded. It can be seen from Figure 20 that without the high-pass filter loaded, the roll angle of the vehicle body fluctuates around 0, and the vehicle body basically remains level. However, from Figure 21a, it can be seen that the equivalent suspension servo actuator cylinder on the right side of the rear axle has been retracted to the shortest position, and it remains at the shortest position during the subsequent driving. However, after loading the high-pass filter, the situation changes. It can be seen from Figure 20 and Figure 21b that the part with slow change frequency in the body roll angle has basically been filtered out, and the displacement of each equivalent suspension servo actuator cylinder is within the stroke range.

On the basis of the above test results and analysis, it can be seen that the high-pass filter can effectively solve the problem of displacement saturation of suspension servo actuator cylinder when the vehicle passes through the ramp road.

## 5. Discussion and Conclusions

In this paper, the multi-axle emergency rescue vehicle was regarded as equivalent to a 3-DOF parallel mechanism by grouping and interconnecting the suspension system units of the whole vehicle, and an ASCS based on IMU was proposed. Taking the three-axle emergency rescue vehicle as the research object, an experimental platform integrating a hydro-pneumatic suspension system and an active suspension system was built, and a gravel road experiment and slope road experiment were carried out. The experimental results for the gravel road show that, compared with hydro-pneumatic suspension, the active suspension control strategy proposed in this paper can effectively reduce the fluctuation of body attitude and significantly improve the handling stability of the vehicle. At the same time, the vertical acceleration, roll angle acceleration and pitch angle acceleration of active suspension are reduced by 25.74%, 21.22% and 22.28%, respectively, compared with the hydro-pneumatic suspension. The experimental results for the slope road show that the high-pass filter is able to remove the part of the vertical attitude that changes slowly when the vehicle is driving on a gentle slope, effectively solving the problem of displacement saturation for the suspension servo actuator cylinders.

Compared with existing control approaches for the vehicles, the ASCS based on IMU proposed in this paper only compensates and regulates the body attitude from the perspective of vehicle kinematics, and eliminates the dependence on the vehicle dynamics model. Additionally, for the control of the actuators, we use position feedback instead of force feedback, which is easily affected by external interference, and this will greatly reduce the difficulty of control. In addition, the multi-axle vehicle suspension units were interconnected in groups, reducing the multi-dimensional control of the active suspension system to three-dimensional control, and effectively reducing the control difficulty of the system.

The active suspension control strategy based on IMU proposed in this paper can not only stabilize the body attitude, but also effectively suppress body vibration, significantly improving the ride comfort and handling stability of the vehicle.

## Figures and Tables

**Table 1:** Single wishbone suspension structural parameters of 1/6 vehicle.

Structural Parameters	Values	Structural Parameters	Values
*α*_1_ (∠ODB)	1.54 (rad)	*l_OB_* (line segment OB)	※ (mm)
*α_2_* (∠AOB)	※ ^1^ (rad)	*l_OD_* (line segment OD)	※ (mm)
*α*_3_ (∠DOB)	※ (rad)	*l_OO_*_’_ (line segment OO′)	※ (mm)
*α*_4_ (∠AOE)	0.18 (rad)	*l_AB_* (line segment AB)	573.69 (mm)
*α*_5_ (∠ODO′)	1.09 (rad)	*l_BD_* (line segment BD)	443.16 (mm)
*α*_6_ (∠EOD)	※ (rad)	*l_O’D_* (line segment O′D)	364.28 (mm)
*α*_7_ (∠BOO′)	※ (rad)	*l_OF_* (line segment OF)	431.17 (mm)
*l_OA_* (line segment OA)	1241.16 (mm)	*l_O’C_* (line segment O’C)	683.25 (mm)

^1^ Symbol ※ indicates that the parameter is a non-fixed value.

## Data Availability

Not applicable.
